# Few serum proteins mediate APOE’s association with dementia

**DOI:** 10.1371/journal.pone.0172268

**Published:** 2017-03-14

**Authors:** Donald R. Royall, Safa Al-Rubaye, Ram Bishnoi, Raymond F. Palmer

**Affiliations:** 1 Department of Psychiatry, the University of Texas Health Science Center, San Antonio, Texas, United States of America; 2 Department of Medicine, the University of Texas Health Science Center, San Antonio, Texas, United States of America; 3 Department of Family and Community Medicine, the University of Texas Health Science Center, San Antonio, Texas, United States of America; 4 South Texas Veterans’ Health System Audie L. Murphy Division Geriatric Research Education and Clinical Care Center, San Antonio, Texas, United States of America; 5 Department of Psychiatry, the Medical College of Georgia, Augusta, Georgia, United States of America; Universiteit Antwerpen, BELGIUM

## Abstract

The latent variable “δ” (for “dementia”) appears to be uniquely responsible for the dementing aspects of cognitive impairment. Age, depression, gender and the apolipoprotein E (APOE) e4 allele are independently associated with δ. In this analysis, we explore serum proteins as potential mediators of APOE’s specific association with δ in a large, ethnically diverse longitudinal cohort, the Texas Alzheimer’s Research and Care Consortium (TARCC). APOE was associated only with C-Reactive Protein (CRP), Adiponectin (APN) and Amphiregulin (AREG), although the latter two’s associations did not survive Bonferroni correction for multiple comparisons. All three proteins were associated with δ and had weak potential mediation effects on APOE’s association with that construct. Our findings suggest that APOE’s association with cognitive performance is specific to δ and partially mediated by serum inflammatory proteins. The majority of APOE’s significant unadjusted effect on δ is unexplained. It may instead arise from direct central nervous system effects, possibly on native intelligence. If so, then APOE may exert a life-long influence over δ and therefore *all-cause* dementia risk.

## Introduction

The latent variable ‘δ” is a dementia phenotype specifying “the cognitive correlates of functional status”. δ appears to be chiefly, if not uniquely, responsible for observed dementia severity [1–2]. Because δ is a fraction of Spearman’s general intelligence factor “*g*” [3], δ’s strong and specific association with dementia (across diagnoses) [1] constrains that syndrome to the pathophysiology of “intelligence”, and potentially to a restricted set of biomarkers.

Age, depression, and the apolipoprotein E (APOE) e4 allele are independently associated with δ [4]. Thus, their associations with both clinical dementia status and with dementia conversion risk may also be constrained to biological processes that affect intelligence. Those processes do not necessarily involve neurodegeneration. Age’s association with δ has been shown to be fully mediated by *a paucity* of neurodegenerative changes in pathologically confirmed AD cases [5]. Additionally, even in their aggregate, these dementia risks explain a minority of δ’s variance. Thus, observed dementia status must be largely determined by age and APOE independent factors.

We have found the majority of δ’s variance to be associated with a large number of pro- and anti-inflammatory serum protein biomarkers, independently of age, depression and APOE [4, 6–8]. If those biomarkers are determinants of neurodegeneration, then age, depression, and APOE may modulate how much neurodegeneration is required to achieve a demented state (i.e., a dementing d-score). Such a finding might explain reports of “cognitive reserve”, and specifically its association with native intelligence [9].

In this analysis, we combine SEM with longitudinal data from the Texas Alzheimer’s Research and Care Consortium (TARCC) to explore more than 100 serum proteins as potential mediators of APOE’s specific association with δ. Our model is constructed such that any significant mediator of APOE’s effect on prospective δ scores can be interpreted causally. Thus, they may offer targets for the remediation of APOE-specific cognitive impairments. However, we predict that APOE’s effects will not be mediated by pro-inflammatory serum proteins. Instead, we note that APOE has been associated with childhood cognitive performance, intelligence testing, and Spearman’s *g* [10]. Thus, APOE’s effects on cognitive performance may be incurred early in life. If so, then APOE may simply alter the baseline from which subsequent neurodegeneration plays out its effects.

## Materials and methods

### Subjects

Subjects included n = 3385 TARCC participants, including 1240 cases of Alzheimer’s Disease (AD), 688 “Mild Cognitive Impairment “(MCI) cases, and 1384 normal controls (NC). Each underwent serial annual standardized clinical examinations, culminating in a consensus clinical diagnosis of NC, MCI or AD. Institutional Review Board approval was obtained at each site and written informed consent was obtained from all participants.

δ’s Indicators included Logical Memory II (LMII) [11], Visual Reproduction I (VRI) [11], the Controlled Oral Word Association (COWA) [12], Digit Span Test (DST) [11] and Instrumental Activities of Daily Living (IADL) [13]. All tests were available in Spanish translation. The latent variables’ indicators were not adjusted for this analysis. The resulting δ homolog was validated by its association with dementia severity, as measured by the Clinical Dementia Rating Scale sum of boxes (CDR) [14] and by Receiver Operating Curve (ROC) analysis.

TARCC’s methodology has been described elsewhere [15]. Serum samples were sent frozen to Rules-Based Medicine (RBM) in Austin, TX. There they were assayed without additional freeze-thaw cycles. RBM conducted multiplexed immunoassay via their human multi-analyte profile (human MAP). A complete listing of the biomarker panel we employed is available at http://www.rulesbasedmedicine.com.

We ran all RBM analyses in duplicate and discarded data when the duplicate values differed by > 5%. All values recorded by RBM as “LOW” were recorded and analyzed. If more than 50% of the samples for a given analyte were recorded as “LOW”, all readings for that analyte were dropped. If less than 50% of the analytes were recorded as “LOW”, the LOW values were recorded as the least detectable dose (LDD) divided by two. Raw biomarker data were inspected to ascertain their normality. Data points beyond 3.0 standard deviations (SD) about the mean were labeled as “outliers” and deleted. Logarithmic transformation was used to normalize highly skewed distributions. The data were then standardized to a mean of zero and unit variance.

### Covariates

All observed measures in the structural models were adjusted for age, education, ethnicity, gender, homocysteine (HCY), and hemoglobin A1c (HgbA1c). Measurements of HCY, HgbA1c and APOE ε4 genotyping were performed in the Ballantyne laboratory at the Baylor College of Medicine. HgbA1c was measured in whole blood by the turbidimetric inhibition immunoassay (TINIA). HCY was measured in serum using the recombinant enzymatic cycling assay (i.e., Roche Hitachi 911).

### APOE genotyping

APOE genotyping was conducted using standard polymerase chain reaction (PCR) methods [16]. APOEε4 status was coded dichotomously based on the presence or absence of an ε4 allele. TARCC’s RBM biomarkers exhibit significant batch effects. Therefore, each biomarker was additionally adjusted for dichotomous dummy variables coding batch.

### Statistical analyses

#### Analysis sequence

This analysis was performed using Analysis of Moment Structures (AMOS) software [17]. The maximum likelihood estimator was chosen. All observed indicators were adjusted for age, education, ethnicity and gender. Co-variances between the residuals were estimated if they were significant and improved fit.

We used the ethnicity equivalent δ homolog (“dEQ”) as previously described [4]. That homolog has been reported to 1) have excellent fit (i.e., χ^2^/df = 181/24, p < 0.001; CFI = 0.97; RMSEA = 0.05), 2) have acceptable factor determinacy by Grice’s Method [18], 3) exhibit factor equivalence across ethnicity, 4) to be strongly correlated with dementia severity as measured by the CDR (r = 0.99, p <0.001) and 5) to exhibit an AUC of 0.97 (CI: 0.97–0.98) for the discrimination between AD cases and controls (in Wave 2 TARCC data). For the purposes of this analysis, dEQ was again constructed in Wave 2 data, but without any covariates, specifically age, ethnicity, GDS, gender, HCY, HGbA1c and APOE ε4 burden.

dEQ and g’ factor weights were applied to Wave 2 observed data to generate Wave 2 dEQ and g’ composite scores (i.e., dEQ w2 and g’ w2, respectively). g’ is dEQ’s residual in Spearman’s *g*. The composite scores were used as observed outcomes in models of a baseline APOE ε4 allele’s direct association with covariate adjusted Wave 2 dEQ.

**Fig 1:**
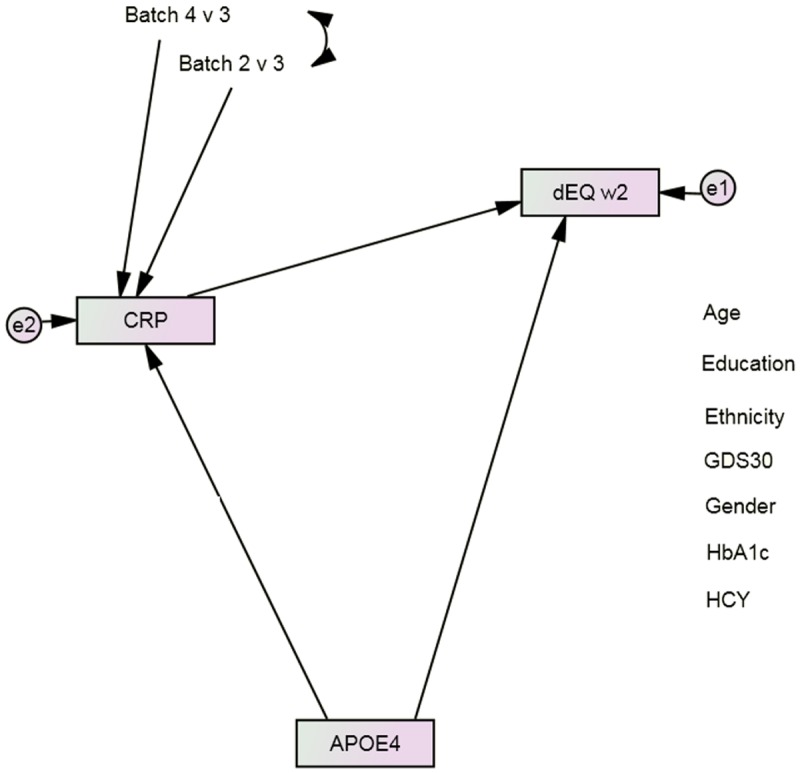
APOE’s direct association with future dementia severity, as measured by dEQ. APOE = apolipoprotein ε4 status; CFI = Comparative Fit Index; GDS = Geriatric Depression Scale; HCY = serum homocysteine; HgbA1c = serum hemoglobin A1c; IGF-BP2 = Insulin-like Growth Factor Binding Protein 2; RMSEA = Root Mean Square Error of Association. *All observed variables except APOE are adjusted for age, education, ethnicity, gender, GDS, HCY, and HgbA1c (paths not shown for clarity). The covariates are densely intercorrelated.

Next, we constructed a longitudinal mediation model in SEM (Fig 1). Such models can arguably be interpreted causally [19]. Path “a” represents the APOE ε4 allele’s direct association with Wave 2 dEQ scores. Path “b” represents the biomarker’s independent effect on dEQ, measured at Wave 1. When both were significant, we considered path “c”. Bonferroni correction to p <0.001 was used to offset the potential for Type 2 error after multiple comparisons. The biomarker’s mediation effect on the APOE ε4 allele’s direct association can then be calculated by MaKinnon’s method [20].

The mediation models were constructed in a randomly selected subset of TARCC participants, comprising approximately 50% of the subjects (i.e., Group 1: n = 1691). As a test of each model’s generalizability to the remainder (n = 1694), each mediation path’s significant direct association was constrained across the two groups, and model fit compared across constrained and unconstrained conditions [21–22]. Mediation effects were calculated in the constrained models.

#### Missing data

We used the newest instance of TARCC’s dataset (circa 2016). The entire dataset was employed. Clinical diagnoses were available on 3385 subjects, 2861 of whom had complete data for δ’s cognitive indicators and covariates. Modern Missing Data Methods were automatically applied by the AMOS software [23]. AMOS employs Full information Maximum Likelihood (FIML) [24–25]. Only the ROC analyses, performed in Statistical Package for the Social Sciences (SPSS) [26], were limited to complete cases.

#### Fit indices

Fit was assessed using four common test statistics: chi-square, the ratio of the chisquare to the degrees of freedom in the model (CMIN /DF), the comparative fit index (CFI), and the root mean square error of approximation (RMSEA). A non-significant chisquare signifies that the data are consistent with the model [27]. However, in large samples, this metric conflicts with other fit indices (insensitive to sample size) show that the model fits the data very well. A CMIN/DF ratio < 5.0 suggests an adequate fit to the data [28].The CFI statistic compares the specified model with a null model [29]. CFI values range from 0 to 1.0. Values below 0.95 suggest model misspecification. Values approaching 1.0 indicate adequate to excellent fit. An RMSEA of 0.05 or less indicates a close fit to the data, with models below 0.05 considered “good” fit, and up to 0.08 as “acceptable”[30]. All fit statistics should be simultaneously considered when assessing the adequacy of the models to the data.

## Results

The demographic characteristics of TARCC’s sample are presented in Table 1. The unadjusted wave 2 dEQ achieved a high AUC for the discrimination between AD cases and NC (AUC = 0.953; CI: 0.946–0.960). g’s AUC for the same discrimination was at a near chance level [AUC = 0.536 (CI: 0.514–0.558)]. This is consistent with past findings, across batteries, in this and other cohorts.

**Table 1:** Descriptive statistics.

Variable	N	Mean (SD)
**Age** (observed)	3381	70.88 (9.48)
**APOE e4 alleles** (1 = e4+, n = 1223)	3154	0.39 (0.49)
**CDR (Sum of Boxes)**	3306	2.42 (3.35)
**COWA**	3381	8.41 (3.49)
**DIS**	3381	8.89 (3.01)
**EDUC** (observed)	3381	13.24 (4.25)
**Ethnicity** (1 = MA, n = 1189)	3381	0.36 (0.47)
**GDS**_**30**_ (observed)	3005	5.60 (5.25)
**Gender** (♂ = 1, n = 1281)	3312	0.39 (0.49)
**IADL (Summed)**	3381	10.48 (4.52)
**MMSE**	3311	25.52 (4.76)
**WMS LM II**	3381	8.05 (4.30)
**WMS VR I**	3381	7.88 (3.68)
**Complete Cases**	2861	

CDR = Clinical Dementia Rating scale; COWA = Controlled Oral Word Association Test; DIS = Digit Span Test; GDS = Geriatric Depression Scale; IADL = Instrumental Activities of Daily Living; MMSE = Mini-mental State Exam; SD = standard deviation; WMS LM II = Weschler Memory Scale: Delayed Logical Memory; WMS VR I = Weschler Memory Scale: Immediate Visual Reproduction.

The Base Model fit well [χ^2^ = 84.80 (11), p <0.001; CFI = 0.977; RMSEA = 0.045]. Independently of the covariates (i.e., age, education, ethnicity, gender, GDS scores, HCY, and Hgb A1c), possession of an APOE ε4 allele was significantly directly associated with Wave 2 dEQ (r = -0.25, p<0.001), but not with the Wave 2 g’ composite (r = -0.02, p = 0.21). g’ was then dropped from consideration. The APOE ε4 allele’s significant association with Wave 2 dEQ scores was in a negative direction suggesting an adverse effect on observed cognitive performance.

The mediation models also fit well [e.g., Adiponectin (APN): χ^2^ = 168.65 (17), p < 0.001; CFI = 0.965; RMSEA = 0.051; Amphiregulin (AREG): χ^2^ = 121.60 (17), p < 0.001; CFI = 0.980; RMSEA = 0.043; C-Reactive Protein (CRP): χ^2^ = 168.58 (17), p < 0.001; CFI = 0.964; RMSEA = 0.051 (Fig 1)]. Regardless, only CRP achieved a statistically significant mediation effect after Bonferroni correction for multiple comparisons (Table 2). CRP appeared to mediate 8.1% of the APOE ε4 allele’s direct effect on δ (z = 3.10, p = <0.001). CRP’s effect replicated across both random subsets [χ^2^ difference = 1.9 (3), p = 0.50].

**Table 2:** Potential mediators of APOE e4’s-specific cognitive effects.

1. Adiponectin (APN)*
2. Amphiregulin (AREG)*
3. C Reactive Protein (CRP)

*Does not survive Bonferroni correction to <0.001.

Two additional serum proteins, APN, and AREG approached significance. Both failed to survive Bonferroni correction, due to relatively weak associations with the APOE ε4 allele along path c (p = 0.008 and 0.004 respectively). APN might otherwise have mediated 5.4% of the APOE ε4 allele’s direct effect (z = -2.52, p <0.001). APN’s potential mediation effect replicated across random subsets [χ^2^ difference = 5.6 (3), p = 0.10].

AREG might otherwise have mediated 7.2% of the APOE ε4 allele’s direct effect (z = -4.54, p ≤ 0.001). AREG’s potential mediation effect replicated across random subsets [χ^2^ difference = 3.2 (3), p = 0.25]. There were no other APOE ε4 allele-associated proteins. Table 3 presents the APOE ε4 allele-independent dEQ biomarkers. Table 4 lists biomarkers that were related neither to the APOE ε4 allele, nor to dEQ.

**Table 3:** APOE-independent dEQ biomarkers (unrelated to APOE by Path c).

1. Agouti-Related Protein (AgRP)
2. alpha1-antitrypsin (A1AT)
3. alpha2-macroglobulin (α2M)*
4. alpha-Fetoprotein (α-FP)
5. angiopoetin-2N
6. Angiotensin Converting Enzyme (ACE)
7. angiotensinogen
8. apolipoprotein A1(APOA1)
9. Apolipoprotein CIII (APOCIII)
10. AXL
11. Betacellulin
12. Bone Morphogenic Protein 6
13. Brain-Derived Neurotrophic Factor (BDNF)
14. CD40
15. Cancer Antigen 125 (CA 125)
16. Cancer Antigen 19–9 (CA 19–9)
17. Compliment 3 (C3)
18. Connective Tissue Growth Factor (CTGF)
19. Cortisol
20. Creatinine Kinase-MB (CK-MB)
21. Eotaxin-3
22. Epidermal Growth Factor (EGF)
23. Epidermal Growth Factor Receptor 1 (EGFR)
24. Epiregulin (EREG)
25. Factor VII
26. FAS
27. FAS-Ligand (FAS-L)
28. Follicle stimulating hormone (FSH)
29. Glutathione S-Transferase
30. Granulocyte Colony Stimulating Factor (GCSF)
31. Heparin-binding EGF-like growth factor (HB-EGF)
32. Hepatocyte Growth Factor (HGF)
33. Immunoglobulin A
34. Immunoglobulin M
35. Insulin
36. Insulin-like Growth Factor-1 (IGF-I)
37. Insulin-like Growth Factor-Binding Protein 2 (IGF-BP2)*
38. Interferon-gamma*
39. Interleukin 1 receptor antagonist (IL-1ra)
40. Interleukin 3 (IL-3)
41. Interleukin 5 (IL-5)
42. Interleukin 7 (IL-7)
43. Interleukin 8 (IL-8)
44. Interleukin 10 (IL-10)*
45. Interleukin 12-p40 (IL-12p40)*
46. Interleukin 13 (IL-13)*
47. Interleukin 15 (IL-15)†
48. Interleukin 16 (IL-16)
49. Lipoprotein a
50. Luteinizing Hormone (LH)
51. Macrophage Inflammatory Protein type 1 alpha (MIP-1a)
52. Macrophage Inflammatory Protein type 1 beta (MIP-1b)
53. Matrix Metalloproteinase type 3 (MMP-3)
54. Monocyte Chemotactic Protein type 1 (MCP-1)
55. Myoglobin (MyG)
56. Pancreatic Polypeptide (PP)
57. Plasminogen Activator Inhibitor type 1 (PAI-1)
58. Platelet-Derived Growth Factor (PDGF)
59. Progesterone
60. Prolactin (PRL)*
61. Prostate Specific Antigen (PSA)
62. Pulmonary and Activation-Regulated Chemokine (PARC)
63. Resistin
64. S100b
65. Serum Amyloid P (SAP)
66. Serum Glutamic Oxaloacetic Transaminase (SGOT)
67. Soluable Advanced Glycosylation End Product-Specific Receptor) (sRAGE)
68. Sortilin
69. Stem Cell Factor (SCF)*
70. Tenascin C
71. Testosterone
72. Thrombopoietin (THPO)*
73. Thrombospondin-1 (THBS1)
74. Thyroxine Binding Globulin (TBG)
75. Tissue Factor (TF)
76. Tissue Growth Factor alpha (TGF-α)
77. Tissue Inhibitor of Metalloproteinase type 1 (TIMP-1)
78. **Tumor Necrosis Factor-Related Apoptosis-Inducing Ligand Receptor 3** (TRAIL-R3)
79. Tumor Necrosis Factor alpha (TNF-α)*
80. Vascular Cell Adhesion Molecule type 1 (VCAM-1)
81. Vitamin D Binding Protein (VDBP)††
82. Vascular Endothelial Growth Factor
83. von Willebrand Factor*

*Previously recognized δ biomarkers in Non-Hispanic White TARCC participants only (Royall & Palmer, 2015).

Previously recognized ethnicity adjusted δ biomarkers (†Bishnoi, Palmer & Royall, 2015a, ††2015b).

**Table 4:** Unrelated biomarkers.

1. Apolipoprotein H (apoH)
2. beta2-macroglobulin (b2M)*
3. B Lymphocyte Chemoattractant (BLC)
4. Carcinoembryonic antigen (CEA)
5. CD40 Ligand
6. Chromogranin A
7. ENA-78 (ENA-78)
8. EN-RAGE (EN-RAGE)
9. Eotaxin
10. Fatty Acid Binding Protein (FABP)
11. Ferritin
12. fibrinogen
13. GRO alpha (GROa)
14. Growth Hormone
15. Haptoglobin
16. Human CC Cytokine (HCC-4)
17. I-309
18. Immunoglobulin E
19. Intercellular Adhesion Molecule, type 1 (ICAM-1)
20. Interleukin 8 (IL-8)
21. Interleukin 18 (IL-18)
22. Leptin
23. Macrophage Derived Chemokine (MDC)
24. Macrophage Migration Inhibitory Factor (MMIF)
25. Prostatic Acid Phosphatase (PAP)
26. RANTES
27. Sex Hormone Binding Globulin (SHBG)
28. Thyroid Stimulating Hormone (TSH)
29. Tumor Necrosis Factor beta (TNFb)
30. Tumor Necrosis Factor receptor type II (TNF-RII)

*Previously recognized δ biomarker in Non-Hispanic TARCC participants only (Royall & Palmer, 2015) (i.e., unconfirmed as a biomarker of dEQ in this ethnicity adjusted analysis. Regardless, shows a trend: r = 0.08, p = 0.02).

## Discussion

We have surveyed more than 100 potential mediators of the APOE ε4 allele’s specific and significant association with δ. Our sample size was large, and we were powered to detect even statistically weak effects. All our findings have been replicated in random subsets of TARCC’s data. We also replicate all but one of our previously observed APOE independent associations with δ [and that exception, beta2-microglobulin (b2M), shows a trend (Table 2)], even though 1) our sample size has increased over time, 2) we are using a new δ homolog, 3) the biomarkers are being used to predict future cognitive performance, and 4) the prior associations were obtained using raw biomarker data while these employ normalized variables. All the other significant biomarkers in Table 2 represent newly identified δ-related serum protein biomarkers.

We have identified three classes of proteins: 1) potential mediators of the APOE ε4 allele’s significant direct effect on δ, 2) APOE independent predictors of δ, and 3) proteins unrelated to either the APOE ε4 allele or δ. Only three serum proteins were possibly related to the APOE ε4 allele, and all were associated with δ.

These observations may help clarify APOE’s role in cognitive function. First, although the APOE ε4 allele has been associated with *g* and *g* is thought to be highly heritable [10], our findings suggest that the ε4 allele’s effect is limited to δ and not g’, i.e., δ’s residual in Spearman’s *g*. APOE may therefore modulate a specific fraction of intelligence. δ in turn has been associated with the DMN [31]. APOE’s effect on DMN structure and function has not been well studied, but the ε4 allele has been associated consistently with β-amyloid (Aβ) deposition [32]. Aβ has also been co-localized with the DMN [33]. Thus, Aβ deposition in the DMN might mediate APOE’s association with dementia, and that association may manifest as a disruption of intelligence, not domain-specific cognitive performance. This hypothesis cannot be tested in TARCC’s data.

Second, δ has been shown to be “agnostic” to dementia’s etiology [1]. APOE’s specific association with δ suggests it may have a role in determining *all cause* dementia risk, not just AD risk. Thus, APOE ε4 burden lowers age of onset across diagnoses and has been implicated as a cognitive determinant in multiple disorders [34].

This may be the first demonstration of any serum protein’s mediation effect on the APOE ε4 allele’s association with either dementia, or with observed cognitive performance. Ironically, the apoE protein itself has been shown to predict future dementia, independently of APOE genotype [35]. The fact that our model is longitudinal favors a causal role for these proteins as potential mediators of APOE’s effect on δ. Only CRP was identified as an unambiguous mediator of APOE’s effect. APN and AREG approached significance. None of these proteins’ associations with δ had been recognized in our prior work, which has been adjusted for APOE ε4 burden.

All potential mediation effects were small, and their associations with the APOE ε4 allele were statistically weak. Our ability to detect weak effects is an expression of TARCC’s large sample size. Regardless, their weak associations replicated across two random subsets of the cohort, and are probably not artifacts. Plasma CRP levels have been associated with an “inflammation-specific AD polygenic risk index” [36]. That finding also implicates CRP as a possible mediator of AD genetic risk.

Moreover, CRP’s weak effect on δ is not likely to be clinically trivial. ε4 appears to more than double 5yr prospective dementia conversion risk in TARCC, independently of multiple covariates. That association is fully attenuated by CRP [37].

The adverse effects serum CRP levels on observed cognitive performance have been reported to be moderated by APOE. CRP’s effect is often reported to occur in the absence of an ε4 allele [38–39]. Our findings clarify that CRP has a positive (salutary) effect on dEQ. However, CRP levels are lowered in the presence of an ε4 allele (by path c). This finding is also consistent with previous studies, which show lower CRP levels in ε4 carriers across multiple populations [40–42].

Serum CRP is lowered by the use of statins [43]. Additionally, hypercholesterolemia may augment ε4’s adverse effect on cognition [42]. Two limitations to our analyses are that we did not consider the effects of either statin use or serum cholesterol in these models. Regardless, lowering CRP still further in ε4 carriers might be expected to have adverse effects on dementia risk, given our present findings. This may explain paradoxical reports of adverse cognitive declines associated with statin use. Post-marketing reports have led to a Food and Drug Administration (FDA) caution against the use of statins by the elderly [44]. Although such anecdotal reports have been difficult to confirm, most investigators approach this task through observed cognitive measures and /or domain-specific indices. Our findings suggest that the effects of statins should be approached from the perspective of general intelligence.

To our knowledge, this is the first demonstration of a potential association between APOE and either APN or AREG. However, APN has previously been associated with prospective cognitive decline in Mild Cognitive Impairment (MCI), and that effect was fully attenuated by APOE adjustment, suggesting an association [45].

AREG, beta-cellulin (BTC), Epidernal Growth Factor (EGF), the Epidermal Growth Factor Receptor 1 (EGFR), Epigen (EPGN), Epiregulin (EREG), Heparin-binding EGF-like growth factor (HB-EGF), and the Neuregulins 1–4 are members of the EGF family of serum proteins [46]. EGF, EGFR, EREG, and HB-EGF were all predictors of δ in these data (Table 2), but none were associated with the APOE ε4 allele. These findings implicate the EGF family of serum proteins as potential modulators of dementia severity, independently of APOE genotype.

However, the above findings are overshadowed by our failure to identify additional potential mediators, as we had originally predicted. That failure was unlikely to reflect statistical power, as multiple δ-related proteins were confirmed by this analysis (Table 2). Nor is it likely to reflect our coding of ε4 allele burden, which was significantly associated with δ. While our findings are necessarily limited to the proteins available in TARCC’s panel, which is neither exhaustive nor rationally selected, they suggest that APOE’s significant association with δ is largely independent of pro-inflammatory serum proteins, as well as all of δ’s previously identified serum protein biomarkers.

Alternatively, APOE’s effects might be limited to the *central* nervous system (CNS), and thus escape detection by our analysis of peripheral blood-based biomarkers. APOE’s association with δ has been shown to be fully mediated by AD-specific neurodegenerative lesions [47], and to contribute to Braak stage [48]. Its association with *g* also appears to be partially mediated by integrity in white matter tracts [10]. AREG has been shown to be an independent mitogen of adult neural stem cells [49], and might also contribute to CNS effects independently of its serum protein levels.

δ’s intercept and slope (Δδ) contribute independently to future dementia severity, and together they explain the vast majority of its variance [1–2]. Regardless, all of δ’s serum protein biomarkers to date appear to be associated with δ’s intercept, and not its slope, in longitudinal analyses (e.g., [8]). Similarly, the presence of an APOE ε4 allele is associated with baseline cognitive performance in older persons, but not its rate of change [50–51]. If APN, AREG and CRP are also related to future d-scores through δ’s intercept, then they may “trigger” APOE-related dementing processes rather than prosecute them.

δ's extraction from general intelligence and *g*’s “indifference” to its indicators further constrain APOE’s effects on δ to an association with intelligence. Native intelligence may influence dementia risk from a very early age by fixing in advance the extent to which an acquired dementing illness has to progress before a dementing δ score is achieved. “General cognitive function” has recently been associated with four genes, including APOE [52]. Thus, early insults to δ may increase the risk of dementia conversion independently of later insults, and /or hasten its age of onset (i.e., the age at which a dementing d-score is achieved). This may explain how the ε4 allele advances the average of age at onset of AD [53] without effecting longitudinal declines in cognitive performance [8].

Possession of an ε4 allele is associated with altered DMN connectivity in cognitively normal elderly [54], and young adults [55], and has even been shown to modulate responses to air pollution in children [56], suggesting very early pre-clinical effects by an Aβ independent mechanism(s). That APOE’s effect may occur in advance of acquired illness could also explain our failure to associate APOE with serum biomarkers, especially since they have been measured proximally to δ scores. If APOE’s effects on cognitive performance are incurred early in life, they may simply alter the field on which the game of neurodegeneration is later played. This again suggests that APOE should be a risk for all-cause dementia, and not just AD.
